# Proof-of-concept for intervention to prevent post-operative ileus in patients undergoing ileostomy formation

**DOI:** 10.1186/s13741-022-00257-0

**Published:** 2022-07-12

**Authors:** Anya L. Greenberg, Yvonne M. Kelly, Ankit Sarin, Madhulika G. Varma

**Affiliations:** 1 School of Medicine, University of California, San Francisco, San Francisco, CA USA; 2 Department of Surgery, University of California, San Francisco, San Francisco, CA USA

**Keywords:** Post-operative ileus, Ileostomy formation, Fluid balance, Intervention for post-operative ileus

## Abstract

**Background:**

Preventing post-operative ileus (POI) is important given its associated morbidity and increased cost of care. The authors’ prior work showed that POI in patients with newly created ileostomies is associated with a post-operative day (POD) 2 net fluid balance of > + 800 mL. The purpose of this study was to conduct an initial assessment of the efficacy of a pilot intervention.

**Methods:**

This is a single-institution, pre–post-intervention, proof-of-concept study conducted on the Colorectal Surgery service at the University of California, San Francisco. The study included 58 procedures with ileostomy formation by board-certified colorectal surgeons between August 13, 2020 and June 1, 2021. The intervention included three adjustments to the standard Enhanced Recovery After Surgery protocol: addition of diuresis, delay in advancement to solid food, and earlier stoma intubation. Demographics, intraoperative factors, post-operative fluid balance, and outcomes (POI, post-procedure length of stay [LOS], hospitalization cost, and re-admissions) were compared between patients pre- and post-intervention.

**Results:**

Eight (13.8%) of the 58 procedures in the intervention period were associated with POI vs. a baseline POI rate of 32.6% (*p* = 0.004). Compared to patients without intervention, those with intervention had 67% less odds of POI (OR 0.33, 95% CI 0.15–0.73, *p* = 0.01). This difference remained significant when adjusted for age, gender, body mass index, procedure duration, and operative approach (adjusted OR 0.32, 95% CI 0.14–0.72, *p* = 0.01). Average POD2 stoma output was 0.3 L greater (1.1 L vs. 0.8L; *p* < 0.001) and net fluid balance was 1.8 L lower (+ 0.3 L vs. + 2.1 L; *p* < 0.00001) for these 58 cases. Average post-procedure LOS was 1.9 days lower (5.3 vs. 7.2 days, *p* < 0.001) and direct cost was $5561 lower ($21,652 vs. $27,213, *p* = 0.004), with no difference in 30-day readmissions (*p* = 0.43).

**Conclusions:**

This pilot intervention shows promise for reduction in POI in patients with newly created ileostomies. Additional assessment is needed to confirm these initial findings.

## Background

Preventing post-operative ileus (POI) in patients undergoing colorectal surgery continues to be an area of active research given its association with increased morbidity and cost of care (Peters et al. 2020; Ahmed Ali et al. 2014; Iyer et al. 2009; Asgeirsson et al. 2010; Mao et al. 2019). Enhanced recovery after surgery (ERAS) pathways for intestinal surgery that include measures to minimize POI and its ramifications, such as multimodal pain regimens and early ambulation, have been widely adopted among surgical practices nationally (Chapman et al. 2018; Lobo et al. 2002; Grass et al. 2019). For a variety of reasons, large intestinal operations in which ileostomies are formed predispose patients to increased risk of ileus (Whitehead and Cataldo 2017), perhaps compounded by the increased resistance the bowel experiences as it traverses the abdominal wall. Indeed, it has been shown that ileostomy formation, as well as surgeon-assessed operative difficulty and bowel handling, are independent risk factors for POI (Vather et al. 2015). However, the body of literature evaluating strategies to prevent POI in patients who undergo ileostomy formation is limited.

Fluid management and diet advancement have been recognized as factors that influence POI in patients undergoing abdominal surgery (Gupta and Gan 2016). Yet, there are no standard guidelines for these factors in the context of an ERAS pathway. Identifying evidence-based guidelines in a population at higher risk of ileus, such as patients undergoing ileostomy formation, would be an important step toward addressing and minimizing POI after intestinal surgery and improving quality of care. In previous work, we completed a retrospective study that showed a POI rate of 32.6% among patients undergoing ileostomy formation at our institution and an independent association with POI in patients with ileostomy who had a net positive fluid balance 2 days after surgery of over 800cc (Greenberg et al. 2021). Based on these findings, we decided to pilot an adjustment to our ERAS pathway to include diuresis and delay diet advancement for this patient population as a quality improvement initiative. In this proof-of-concept study, we describe our intervention and report early results from the pilot. This initial assessment of the efficacy of our intervention provides a foundation for a larger future study.

## Materials and methods

This study was approved by the Institutional Review Board at the University of California, San Francisco (UCSF): Study Number 18-26677. Reporting guidelines as outlined in the Standards for Quality Improvement Reporting Excellence (SQUIRE) 2.0 were followed.

### Enhanced recovery after surgery pathway at our institution

An enhanced recovery after surgery (ERAS) pathway was implemented at our institution for all patients undergoing abdominal colorectal surgery in 2014. Prior to implementation of this pilot intervention, the pathway included day-to-day guidance on multimodal pain control, fluid administration, activity level, nutrition, and other nursing treatments (e.g., foley removal, chewing gum, shower) for patients undergoing different colorectal operations. Per our standard ERAS pathway, patients undergoing ileostomy formation received Lactated Ringers in 5% Dextrose solution at 50 ml/h intravenously on post-operative day (POD) 0, which was discontinued on POD1 if patients did not show clinical signs of volume deficit (e.g., tachycardia or orthostatic hypotension, oliguria, or acute elevation in blood urea nitrogen [BUN] or creatinine [Cr] meeting the criteria of acute kidney injury [AKI]). We define tachycardia as > 100 beats per minute; orthostatic hypotension as systolic blood pressure decrease of at least 20 mmHg or diastolic blood pressure decrease of at least 10 mmHg within three minutes of standing; oliguria as urine output < 0.5 mL/kg/h; and AKI as increase in serum creatinine by ≥ 0.3 mg/dL within 48 h, or increase in serum creatinine to ≥ 1.5 times baseline, or urine volume < 0.5 mL/kg/h for 6 h (KDIGO clinical practice guideline for acute kidney injury 2012). Patients were restricted to a clear liquid diet not to exceed 2 L in a 24-h period on POD0 and advanced to a low-residue diet on POD1 if no nausea was present.

### Pilot intervention

Our pilot intervention consisted of three adjustments to this protocol for patients undergoing new ileostomy formation (either end ileostomy or diverting loop ileostomy). The first adjustment was to administer furosemide (Lasix) 10 mg intravenously on POD1 if the patient’s net fluid balance was greater than 500 mL, ileostomy output was less than 1.5 L, and kidney function, as measured by BUN and creatinine, was at the patient’s baseline. The second was a change in diet advancement to a new protocol of restricted clear liquids on POD0, a full liquid diet on POD1, and a low residue diet on POD2. The third was intubation of the stoma with a red Robinson catheter on the morning of POD1 if ileostomy output was < 100 mL since operation. No changes in pain control, activity level, or other nursing treatments were made to the ERAS protocol for these patients as part of our pilot intervention.

**Fig. 1:**
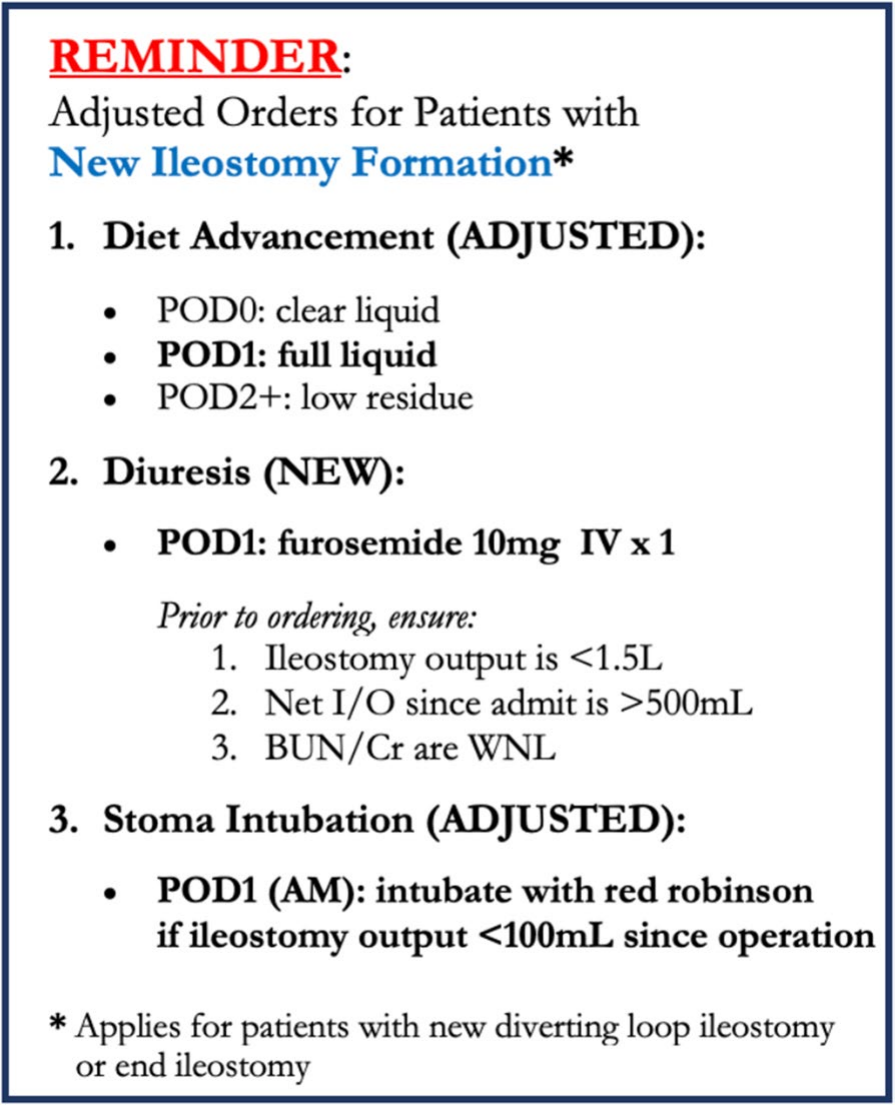
Visual reminder of ERAS adjustment disseminated to clinical staff and posted in team workroom, original

Information about the new protocol was shared with the attendings on the service, residents rotating on the service, and advanced practice providers who are permanently assigned to the colorectal surgery service. To ensure compliance with the changes, we updated our official internal protocol documents circulated to faculty, clinical staff, and trainees rotating on the colorectal surgery service; provided an in-service to faculty and clinical staff on the colorectal surgery service; included a visual reminder (Fig. 1) in the team workroom; and incorporated the new guideline into sign-out between the outgoing and incoming chief surgical resident on the rotation to disseminate to the rest of the team. Furthermore, the nurse practitioners, who are consistent members of the team, were asked to reinforce these protocols. The attending surgeons also continually reinforced the ERAS adjustment with the team and kept track of all patients who participated in the pilot.

### Study population and comparison group

The pilot period included 58 non-emergent procedures between August 13, 2020 and June 1, 2021 that included formation of an ileostomy in which the pilot intervention was employed. The baseline comparator period included all consecutive patients who had elective or urgent (i.e., non-emergent) abdominal colorectal surgery that included formation of an ileostomy by a board-certified colorectal surgeon from July 1, 2015 to June 30, 2020. These were patients on the standard ERAS pathway without pilot intervention. Procedures with ileostomy formation were identified by querying a prospectively-maintained database using current procedural terminology (CPT) codes that included ileostomy formation. Patient charts associated with cases identified through the query were reviewed to confirm they included ileostomy formation.

### Outcome measures

Our primary outcome of interest was the development of POI. Secondarily, we assessed stoma output and net fluid balance as of POD2; post-procedure length of stay [LOS]; direct cost of hospitalization; and 30-day readmissions.

### Study variables

Demographic, intraoperative, and select outcome (LOS, direct cost of hospitalization, and 30-day readmissions) data for both time periods were obtained from an automated ERAS report from our electronic medical record (EMR). Chart review was performed for all ileostomy formation cases in both time periods to identify daily fluid balance, ileostomy output, and whether the patient developed POI. During the patient’s hospitalization, onset of POI was defined clinically by the primary team based on the findings of post-operative nausea and vomiting, abdominal distension, and/or imaging suggesting POI and treated by restricting diet and placing a nasogastric (NG) tube. For the study, patients with POIs were identified by reviewing each patient’s chart to identify documentation of POI or, in the absence of explicit documentation of POI in a patient’s chart, evidence of ileus-specific intervention such as prolonged NPO status (POD3 or longer) or post-operative NG tube placement.

### Data analysis

SAS V9.4 (SAS Institute, Cary, NC, USA) was used to summarize these variables via simple descriptive statistics and compare pilot data to our institution’s baseline data consisting of patients who had an ileostomy formation between July 1, 2015 and June 30, 2020. Two sample *t* tests were used to compare continuous variables and Fisher’s exact tests were used to compare categorical variables. Logistic regressions were used to evaluate the association between POI and covariates.

## Results

This intervention was piloted in 58 procedures (vs. 261 in the baseline period) during which an ileostomy was formed between August 13, 2020 and June 1, 2021. Thirty (52%) of the procedures in the study period were performed for female patients (vs. 46% of those in baseline period; *p* = 0.47) and the mean patient age was 46.2 (vs. 50.4 in the baseline period; *p* = 0.06) (Table 1). Of the 58 procedures in which an ileostomy was formed, 74% were diverting loop ileostomies (vs. 66% in the baseline period) while 26% were end ileostomies (vs. 34% in the baseline period; *p* = 0.28). Types of procedures and indications for surgery can be seen in Tables 2 and 3, respectively.

**Table 1:** Demographics, intraoperative characteristics, post-operative fluid balance, and outcomes of study population compared to baseline

	Baseline *n* = 261	Study period *n* = 58	*p* value
**Demographics**			
Gender			
Female	119 (45.6%)	30 (51.7%)	0.47
Male	142 (54.4%)	28 (48.3%)	
Age (years)	50.4	46.2	0.06
Body mass index (kg/m^2^)	25.4	25.5	0.91
**Intraoperative characteristics**			
Operative approach			
Open	45 (17.2%)	12 (20.7%)	0.41
Laparoscopic	150 (57.5%)	36 (62.1%)	
Robotic	66 (25.3%)	10 (17.2%)	
Ileostomy type			
Diverting loop ileostomy	173 (66.3%)	43 (74.1%)	0.28
End ileostomy	88 (33.7%)	15 (25.9%)	
Procedure duration (min)	289.9	290.8	0.95
**Post-operative characteristics**			
**Two-day ileostomy output (L)**	**0.8**	**1.1**	**< 0.001**
**Two-day post-operative fluid balance (L)**	**2.1**	**0.3**	**< 0.001**
**Outcomes**			
**Post-operative ileus rate**	**85 (32.6%)**	**8 (13.8%)**	**0.004**
**Post-procedure length of stay (days)**	**7.2**	**5.3**	**< 0.001**
**Direct cost**	**$27,213**	**$21,652**	**0.004**
30-day readmissions	46 (17.6%)	7 (12.1%)	0.43

**Table 2:** Types of procedures by study population and comparator group

	Baseline (***n*** = 261)			Study period (***n*** = 58)		
	Ileostomy type		All	Ileostomy type		All
	DLI	End		DLI	End	
Low anterior resection	134	0	134	33	0	33
Total colectomy	0	54	54	0	9	9
Total proctocolectomy^a^	1	32	33	0	3	3
Total proctocolectomy with J-pouch	20	0	20	3	0	3
Proctectomy with J-pouch	18	0	18	5	0	5
Other procedures^a^	0	2	2	2	3	5
**Grand total**	**173**	**88**	**261**	**43**	**15**	**58**

^a^One patient had total proctocolectomy with end ileostomy planned, but because the terminal ileum was tethered by mesenteric tumor, an ileostomy with loop configuration was created and efferent end was stapled off to ensure bowel decompression

^b^Includes completion proctectomy, sigmoidectomy, or right hemicolectomy

*DLI* diverting loop ileostomy, *End* end ileostomy, *J-pouch* ileal pouch-anal anastomosis

**Table 3:** Indications for surgery

	Baseline (***n*** = 261)	Study period (***n*** = 58)
Cancer	140 (53.6%)	33 (56.9%)
Inflammatory bowel disease	96 (36.8%)	21 (36.2%)
Another condition	25 (9.6%)	4 (6.9%)
**Grand total**	**216 (100%)**	**58 (100%)**

Furosemide was administered on POD1 for 44 of these 58 procedures (this includes 4 patients who were taking diuretics at baseline and were given the standard dose according to our protocol on POD1). Of these 44 cases, 11 represented cases in which furosemide was administered to patients who fell outside our guidelines for furosemide administration: nine had POD1 net fluid balance less than +500mL and two had BUN or Cr levels outside of “normal limits” (which is defined by our institutional laboratory as 7–25 for BUN and 0–1.24 for Cr). Of these 44 cases, two had heart rate between 100 and 110 beats per minute and 6 had systolic blood pressure (SBP) between 80 and 100 mmHg within 12 h prior to administration. For two of the six patients with SBP between 80 and 100 mmHg within 12 h prior to administration, SBP was more than 30 mmHg below the pre-operative baseline. Of these 44 cases, no one met definition of AKI and one patient experienced hypotension within 24 h of furosemide administration. Furosemide was not given to anyone with ileostomy output greater than 1.5 L; acute elevation in Cr meeting definition of AKI; heart rate greater than 110; or systolic blood pressure less than 80.

None of the 58 patients in the study period were advanced to a low residue diet before POD2 and stomas of any patient with < 100 mL on POD1 were intubated with a red Robinson catheter. There were no stoma-related complications or aspirations among patients within the study period.

Eight (13.8%) of the 58 procedures in our intervention period were associated with POI vs. a baseline POI rate of 32.6% that was reported in our retrospective study (*p* = 0.004). Compared to patients without intervention, those with intervention had 67% less odds of POI (OR 0.33, 95% CI 0.15–0.73, *p* = 0.01) (Table 4). This difference remained significant when adjusted for age, gender, body mass index (BMI), procedure duration, and operative approach (adjusted OR 0.32, 95% CI 0.14–0.72, *p* = 0.01).

**Table 4:** Odds of post-operative ileus

	OR	95% CI		***p*** value
		Lower	Upper	
Bivariate model	0.33	0.15	0.73	0.01
Multivariable model	0.32	0.14	0.72	0.01

*OR* odds ratio, *CI* confidence interval

Compared to the baseline period, stoma output as of the morning of POD2 for these 58 cases was 0.3 L greater (1.1 L vs. 0.8 L, *p* < 0.001) and average net fluid balance for the same time period was 1.8 L lower (0.3 L vs. 2.1 L, *p* < 0.001). Moreover, the average post-procedure LOS for these patients was 1.9 days lower (5.3 days vs. 7.2 days, *p* < 0.001) and direct cost was $5561 lower ($21,652 vs. $27,213, *p* = 0.004), with no difference in 30-day readmissions (*p* = 0.43). None of the patients in the pilot experienced a complication and no change in renal function was noted that was attributed to the intervention.

## Discussion

In this study, we investigated the efficacy of our pilot intervention in reducing the rate of POI in patients undergoing ileostomy formation as a proof-of-concept for a larger future study. We based our intervention on established principles that fluid management and diet advancement are key factors (Grass et al. 2019) in the development of POI, as well as the critical finding from our prior work that POI is independently associated with a POD2 net fluid balance of greater than 800 mL (Greenberg et al. 2021). The former served as the basis for our intervention conceptually, while the latter served as an evidence-based guideline for a fluid balance goal.

As expected, given the intervention included diuresis, patients in the study period had a significantly lower fluid balance than those in the baseline period. They also had significantly higher ileostomy output than patients in the baseline period. Importantly, our results show the intervention group had a significantly lower rate of POI, post-procedure LOS, and cost of hospitalization with no difference in readmissions compared to the baseline period. This is an important finding given concerns of association between decreased LOS and increased readmissions. Prior work has identified shorter LOS for patients undergoing general and colorectal surgery as a risk-factor for readmission in certain circumstances, such as when patients have multiple post-operative complications (Kohlnhofer et al. 2014; Schneider et al. 2012) or in the setting of very early discharge (≤ 4 days) (Hendren et al. 2011). That LOS in our pilot population decreased without concomitant increase in readmissions is reassuring and suggests that our intervention did not introduce unintended consequences.

**Fig. 2:**
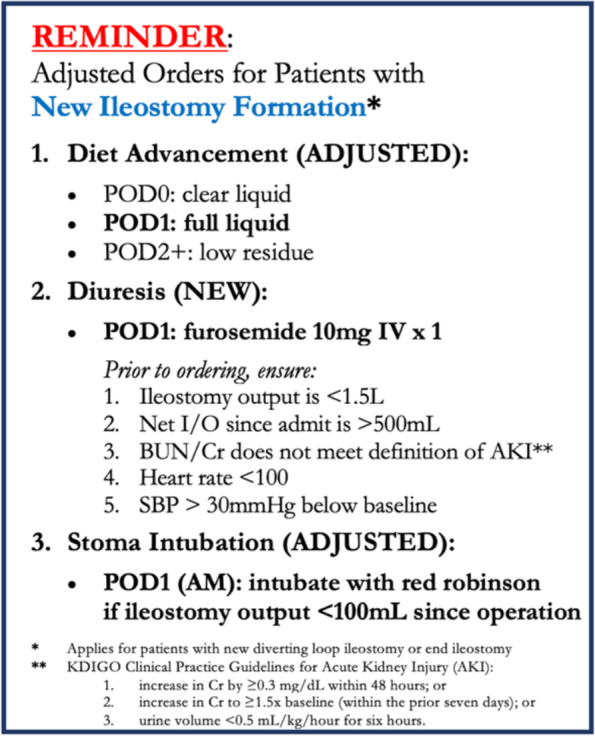
Visual reminder of ERAS adjustment disseminated to clinical staff and posted in team workroom, updated

Though promising, our study revealed important considerations that enabled us to iterate on the pilot intervention. First, our findings elucidated opportunities to clarify and expand our protocol parameters for furosemide administration. While no one who met the definition of AKI received furosemide in the pilot, two patients had BUN or Cr levels outside of our laboratory’s “normal limits”. This prompted us to update our protocol and visual reminder language to reflect the clinically relevant definition of AKI. Similarly, while no one with heart rate above 110 received furosemide, the two patients with heart rate between 100 and 110 within 12 h prior to furosemide administration revealed a gap in our original protocol. Finally, while no patients with SBP below 80 mmHg received furosemide, the two patients who were more than 30 mmHg below their pre-operative baseline revealed another gap in our original protocol. This prompted us to add additional guidelines that furosemide not be administered if heart rate is greater than 100 or if SBP is more than 30 mmHg below the patient’s baseline. These changes in protocol identified from the pilot study resulted in updates to our official service-wide protocol documents and visual reminder (Fig. 2).

Second, our pilot study revealed the need to reinforce specifics of the protocol to members of the colorectal surgery service. Our data analysis revealed that the guidelines (as they were during the pilot period) were followed in most cases. All patients who did not receive furosemide either fell outside the guidelines of the interventions or had signs of hypotension or tachycardia, suggesting that those omissions of furosemide were deliberate rather than inadvertent. However, there were situations in which furosemide was given despite the patient not meeting guideline criteria (e.g., nine patients with POD1 net fluid balance less than + 500 mL). This highlights an opportunity to continually reinforce the protocol to permanent and rotating members of the team to ensure that the specified guidelines are followed for each patient.

While further assessment of the refined protocol as part of a larger study is needed, findings from this pilot show promise that an intervention focused on minimizing bowel edema through diuresis (Godat et al. 2013; Webb et al. 2012) thus avoiding lumen narrowing and preventing mechanical outlet obstruction, and delaying advancement to solid food until patients adapt may have a role in minimizing POI. The tools (e.g., revised visual reminder) and processes (e.g., incorporation into official ERAS protocol, expanded distribution strategy) from this proof-of-concept study could be used to further evaluate the effectiveness of our intervention. An interrupted time-series analysis using segmented linear regression comparing the two time periods designated pre-intervention and post-intervention, with the date of intervention roll-out the start of the post-intervention period, may be used for this assessment.

A limitation in our study is that it is a single-site investigation with a relatively small sample size during the study period. However, this sample size is adequate given our study objective of establishing preliminary efficacy of our intervention as a proof-of-concept for further study. A second limitation is the subjective nature of the POI diagnosis, which relies on a combination of signs, symptoms, and objective data. The retrospective nature of our study may introduce bias into the identification of POI given that standard diagnostic approach was not explicitly defined as part of the study. However, the colorectal surgery service at our institution is comprised of a small, tight-knit group of attendings, with regularly-held service-wide meetings, conferences, and education sessions. This facilitates consistency in the diagnostic data gathering for and documentation of suspected ileus, thus minimizing the potential bias introduced into our retrospective study. In addition, the diagnosis of POI in clinical settings is often subjective and our study is therefore more generalizable to real world scenarios. Finally, a third limitation is the trend toward a significant difference in average age between the study group and the baseline comparator. However, in our prior work spanning a 5-year study period, age was not found to be an independent factor associated with development of POI in patients undergoing ileostomy formation.

## Conclusion

Findings from this proof-of-concept study show promise of an intervention focused on fluid management and diet advancement as a potentially effective strategy for prevention of POI in patients undergoing ileostomy formation. Specifically, administering furosemide on POD 1, intubating stoma early, and delaying intake of solid food by a day is associated with reduced rate of POI in patients with new ileostomies. Additional assessment as part of a larger study is needed to demonstrate efficacy of this intervention.

## Data Availability

The datasets used and/or analyzed during the current study are available from the corresponding author on reasonable request.

## Abbreviations

POI: Post-operative ileus
ERAS: Enhanced recovery after surgery
POD: Post-operative day
BUN: Blood urea nitrogen
Cr: Creatinine
AKI: Acute kidney injury
CPT: Current procedural terminology
LOS: Length of stay
EMR: Electronic medical record
NG: Nasogastric
SBP: Systolic blood pressure
BMI: Body mass index
